# UCHL1 as a novel target in breast cancer: emerging insights from cell and chemical biology

**DOI:** 10.1038/s41416-021-01516-5

**Published:** 2021-09-08

**Authors:** Milon Mondal, Daniel Conole, Jaya Nautiyal, Edward W. Tate

**Affiliations:** 1 Department of Chemistry, Imperial College London, London, UK; 2 Department of Surgery and Cancer, Institute of Reproductive and Developmental Biology, Imperial College London, London, UK

**Keywords:** Breast cancer, Ubiquitylation, Proteomics, Target validation

## Abstract

Breast cancer has the highest incidence and death rate among cancers in women worldwide. In particular, metastatic estrogen receptor negative (ER–) breast cancer and triple-negative breast cancer (TNBC) subtypes have very limited treatment options, with low survival rates. Ubiquitin carboxyl terminal hydrolase L1 (UCHL1), a ubiquitin C-terminal hydrolase belonging to the deubiquitinase (DUB) family of enzymes, is highly expressed in these cancer types, and several key reports have revealed emerging and important roles for UCHL1 in breast cancer. However, selective and potent small-molecule UCHL1 inhibitors have been disclosed only very recently, alongside chemical biology approaches to detect regulated UHCL1 activity in cancer cells. These tools will enable novel insights into oncogenic mechanisms driven by UCHL1, and identification of substrate proteins deubiquitinated by UCHL1, with the ultimate goal of realising the potential of UCHL1 as a drug target in breast cancer.

## Introduction

### The role of estrogen in breast cancer

Breast cancer is the most commonly diagnosed cancer and causes most cancer deaths among women worldwide [1]. The sex hormone estrogen plays a pivotal role in regulating both normal and breast cancer cell proliferation by regulating proteins involved in the cell cycle [2]. Estrogen-regulated breast cancer cell growth involves estrogen binding to estrogen receptor (ER), a transcription factor with two isoforms, ERα and ERβ. On binding to estrogen, ER translocates to the nucleus where it binds to thousands of gene regulatory sites in the genome to regulate expression of gene networks that influence cell growth and proliferation. ERα plays a pivotal role in breast cancer [3], being the predominant isoform expressed in 70–80% of all breast cancers, making it an attractive therapeutic target [4]. ERβ is known for its anti-proliferative activity in breast cancer, and prevents ductal cancer from becoming invasive [4]. Historical breast cancer classification is largely based on the cancer driver activity of ERα along with other hormone receptors, progesterone receptor (PR) and receptor tyrosine kinase HER2/ERBB2. Based on the expression of these proteins breast cancer is classically divided into five subtypes; luminal A (ER+, PR+, HER2−), luminal B (ER+, PR−, HER2+), HER2 positive (ER−, PR−, HER2+) and basal-like and triple-negative breast cancer (TNBC) breast cancers (ER−, PR− and HER2−), which are two similar but distinct subtypes of aggressive breast cancers. ER+ breast cancers initially respond well to endocrine therapy such as estrogen receptor targeting drug tamoxifen [5]; however, one third of metastatic ER+ breast cancer cases gradually lose ER expression and acquire resistance to these agents [6]. ER– breast cancers are more aggressive than ER+ breast cancers [7], and TNBCs are the most aggressive sub-type and also very difficult to treat due to their lack of ER, progesterone receptor and HER2 expression. Metastatic ER− and triple-negative breast cancers have very limited treatment options available and are a leading cause of cancer-related death in women [8–12]. Thus understanding the mechanisms of ER regulation in breast cancers is critical for developing effective therapeutic strategies for their treatment.

### The ubiquitin-proteasome system (UPS) and the deubiquitinase UCHL1

Ubiquitination is the reversible post-translational modification (PTM) of a protein with the small protein ubiquitin (Ub), and while it can have diverse effects on protein function, Ub is best known for its capacity to promote proteasomal degradation [13]. This process is a component of the ubiquitin-proteasome system (UPS) that regulates protein turnover in cells [14]. There are hundreds of enzymes involved in the addition (Ub ligases) or removal (Ub hydrolases) of ubiquitin, and the UPS has emerged as an important source of drug targets in many diseases [15–20]. Deubiquitinases (DUBs) are Ub hydrolases responsible for the removal of Ub PTMs by cleaving the isopeptide bond between the C-terminal glycine of Ub and surface lysine residues on target proteins [14]. Around 100 DUBs have been identified to date, with several having pivotal roles in cancer progression; they are classified into five subcategories based on sequence similarity, four of which encompass papain-like cysteine protease DUBs [21–23], including ubiquitin C-terminal hydrolases (UCH), ubiquitin specific protease (USP), ovarian tumour (OTU) and the Josephin domain, while the Jab1/Mov34/Mpr1 Pad1 N-terminal+ (MPN+) (JAMM) sub-type belongs to the zinc-metalloprotease family. Although UCHs and many USPs have a related 3D architecture and a superimposable catalytic triad, they are distinct in catalytic site amino acid sequence. In UCHs, the catalytic cysteine sits in a narrow groove on the protein surface, which restricts the accommodation of larger side chain residues, leading to increased substrate specificity [21]. Ubiquitin Carboxy-Terminal Hydrolase L1 (UCHL1) is a member of the UCH family of DUBs that is expressed in varied amounts across tissues and tumours, from being among the most abundant proteins in the brain [24–28] to undetectable levels in nasopharyngeal, gastric, colorectal, renal cells and ovarian carcinomas [29–32]. There has been much debate over the role of UCHL1 in cancer, with reports proposing oncogenic [26, 33, 34] or tumour suppressor [29, 35, 36] roles, but a full discussion of these works is not within the scope of the review. Detailed understanding of the mechanism by which UCHL1 is implicated in oncogenesis is currently hampered by a poorly defined set of UCHL1 substrate proteins and a previous lack of pharmacological tools to inhibit UCHL1 activity. Here we will discuss the emerging evidence for the role that UCHL1 plays in the regulation of ER and the Transforming Growth Factor beta (TGF-β) pathway, dysregulation of which impacts the development of aggressive forms of breast cancer (ER− and triple-negative breast cancer) and also development of endocrine resistance. We summarise reports of the first UCHL1 inhibitors and activity-based probes (ABPs) to delineate UCHL1-driven oncogenic mechanisms, and assist in the validation of this potential therapeutic target in cancer.

## Role of UCHL1 in breast cancer

### A novel EGFR/UCHL1/ER axis in ER− breast cancer

**Fig. 1:**
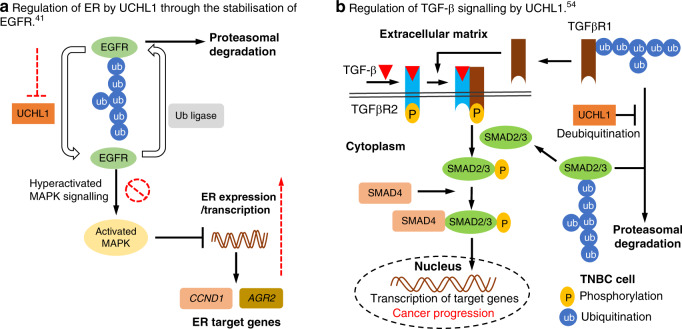
Regulation of ER and TGF-β signalling by UCHL1. **a** Regulation of ER by UCHL1 through the stabilisation of EGFR. EGFR homeostasis is maintained by Ub ligases and DUBs; however, overexpression of UCHL1 tips this balance towards deubiquitination and stabilisation of EGFR, and subsequent hyperactivation of MAPK signalling, thereby downregulating ERα expression and gene transcription. Targeting UCHL1 could promote proteasomal degradation of EGFR, and thus influence ER expression and responses to anti-estrogen therapy [41]. **b** Regulation of TGF-β signalling by UCHL1. UCHL1 is proposed to regulate TGF-β signaling in metastatic breast cancer by deubiquitinating and stabilising TGFβR1 and SMAD2/3 in TNBC and aggressive tumours. TGF-β signalling is induced by TGF-β binding to TGFβR2, which subsequently phosphorylates TGFβR1 which in turn phosphorylates Smad2/3. Phosphorylated Smad2/3 binds Smad4 and the protein complex is translocated to the nucleus to regulate target gene transcription. Knockdown or inhibition of UCHL1 has been proposed to exert anticancer effects by rescuing the ubiquitination and degradation of TGFβR1 and SMAD2/3 [54].

The UPS plays several important roles in regulating ER activity. ER monoubiquitination at the Lys48 residue by ubiquitin ligases leads to enhanced ER stability and transcriptional activity [37]; however, ubiquitin chain growth at this site (polyubiquitination) leads to degradation of ER at the proteasome [38]. On the other hand, DUBs can reverse polyubiquitination by cleaving the ER polyubiquitin chain, leading to ER stabilisation [39]. It was recently suggested that ubiquitin specific protease 7 (USP7) directly deubiquitinates and stabilises ERα in human breast tumour tissue [40]. In contrast, it has been known for some time that UCHL1 mRNA level inversely correlates with ER expression level and is highly associated with the recurrence and invasion in breast cancer patients, although a causal relationship was lacking [28]. In 2020, Chen et al. through various lines of experimental evidence provided a more detailed explanation for this correlation, proposing that UCHL1 deubiquitinates and stabilises Epidermal Growth Factor Receptor (EGFR), which in turn causes suppression of ER transcription [41]. Analysis of UCHL1 and ERα expression in patient-derived breast cancer samples showed that UCHL1 expression was significantly higher in TNBC compared to Luminal A, Luminal B and HER2+, suggesting an association between UCHL1 expression and absence or loss of ER expression. Moreover, survival analysis of the effect of UCHL1 expression in ER+ breast cancer patients with tamoxifen treatment indicated that high expression of UCHL1 was significantly associated with poor prognosis in patients with breast cancer [41]. To investigate further, the authors overexpressed UCHL1 in ER+ cell lines and knocked down UCHL1 in ER− cell lines [41]. The results demonstrated that the overexpression of UCHL1 drastically reduced protein levels of ER and mRNA levels of certain ER target genes, while siRNA knockdown of UCHL1 increased ER expression and induced ER targets (Fig. 1a). Chromatin immunoprecipitation (ChIP) assays showed that UCHL1 knockdown enhanced recruitment of ER to promoter regions of Nuclear Receptor Interacting Protein (NRIP1) and CCND1 genes in ER− breast cancer cells treated with estrogen. In addition, an estrogen response element (ERE) luciferase assay suggested that UCHL1 knockdown increased ER transcriptional activity in ER− cell lines treated with estrogen. Taken together, these results demonstrated that ER restoration by UCHL1 knockdown is functionally active.

It is unlikely that UCHL1 destabilises ER protein directly, since deubiquitination is typically stabilising. In keeping with this hypothesis, the authors showed that proteasome inhibition with MG132 did not abrogate ER down regulation in UCHL1-transfected MCF-7 cells [41]. Rather, UCHL1 overexpression or knockdown decreased or increased ER mRNA levels, respectively, suggesting that UCHL1 regulates ER transcription. As epidermal growth factor receptor (EGFR) is a known suppressor of ER transcription [42] and UCHL1 may modulate EGFR expression [43], Chen et al. chose to explore a possible role for EGFR in modulation of ER expression by UCHL1 using EGFR overexpression or silencing experiments in ER− and ER+ cell lines [41, 42]. EGFR overexpression decreased ER levels in both an ER+ cell line and in an ER− cell line in which ER was induced by UCHL1 knockdown, while EGFR knockdown restored ER expression in an ER+ cell line in which ER expression was downregulated by overexpressing UCHL1 [41]. These results indicate that EGFR has an important role in regulating the UCHL1-mediated effect on ER expression, and proteasome inhibition and immunoprecipitation experiments further supported the hypothesis that UCHL1 regulates EGFR stability in a DUB activity-dependent manner. Overexpression of EGFR is frequently observed in ER− breast cancer, which in turn hyperactivates mitogen-activated protein (MAP) kinase (MAPK) signalling [44–46], which may induce loss of ER gene transcription and expression [42, 47], suggesting that UCHL1 regulates ER stability through EGFR stabilisation and subsequent MAPK hyperactivation. Finally, the authors showed that inhibiting or silencing UCHL1 can sensitise ER− cell lines to anti-estrogen therapy [41]. On the other hand, it has been reported that high expression of UCHL1 in TNBC cell lines facilitates cell invasion through activation of the Akt signalling pathway [48], and correlates with negative ER expression and overall shorter survival of breast cancer patients [49]. While a comprehensive mechanism for the correlation between UCHL1 expression and overall survival of TNBC patients remains to be elucidated, UCHL1 could be a biomarker for breast cancer outcome or treatment decisions, particularly in ER− and TNBC [41].

Even though UCHL1 negatively regulates ER expression through the stabilisation of EGFR and subsequent hyperactivation of MAPK signalling, direct EGFR and/or MAPK silencing or inhibition were not explored by Chen et al. to elucidate their effects on ER expression and endocrine therapy in breast cancer cell lines with modulated UCHL1 expression [41]. Further experiments will therefore be required to identify the potential of combination strategies with UCHL1 inhibitors for the treatment of ER− breast cancer and TNBC patients. Careful selection of these agents will be important, since lung cancer patients who respond initially to a first generation small-molecule tyrosine kinase inhibitor (TKI) such as erlotinib generally develop drug resistance in later stage cancer due to secondary mutations such as EGFR-T790M, which can in turn be treated effectively with a second generation TKI such as gefitinib [50–53]. Therefore, targeting UCHL1 in combination could represent a novel approach to restore ER level in ER– breast cancer and TNBC patients and to de novo sensitise breast cancer patients for endocrine therapy.

### A role for UCHL1 in maintaining transforming growth factor beta (TGF-β) signalling in TNBC

In a separate study using a DUB activity profiling approach Liu et al. identified UCHL1 as a particularly active DUB in TNBC, proposing that UCHL1 also promotes TGF-β-induced TNBC metastasises by deubiquitinating and stabilising TGFβR1 and SMAD2 [54]. Using two DUB activity-based probes (discussed in more detail in the following section), TAMRA-ubiquitin-VME (TAMRA: tetramethylrhodamine, VME: vinyl methyl ester) and Biotin-ubiquitin-VME, the authors identified UCHL1 as the most active DUB across over a large cohort of breast cancer cell lines and aggressive breast cancer tumour tissue samples [54]. Like Chen et al. [41], Liu et al. also observed that UCHL1 activity in TNBC and ER− breast cancer cells is significantly higher compared to non-TNBC and ER+ cells [54]. The authors investigated whether UCHL1 promotes breast cancer metastasis by overexpressing UCHL1 in mCherry MDA-MB-231 cells, which are UCHL1-low, and knocking down UCHL1 in mCherry MDA-MB-436 cells, which are UCHL1-high, and injecting these cells into zebrafish embryos, showing that UCHL1 overexpression or knockdown provokes a stronger or weaker metastatic phenotype, respectively, compared to untransfected control cells [54]. The potential role for UCHL1 in promoting breast cancer invasion and metastasis was further supported in a murine breast cancer xenograft model, where UCHL1 overexpressing groups exhibited increased metastasis compared to UCHL1 knockdown groups.

To explore the mechanism of action by which UCHL1 promotes breast cancer metastasis, Liu et al. [54] investigated UCHL1 depletion in epithelial–mesenchymal transition (EMT), which has previously been shown to play an important role in breast cancer metastasis [55]. The authors identified that knockdown of UCHL1 in MDA-MB-436 cells significantly reduces the expression of mesenchymal markers such as VIMENTIN, SNAIL and SLUG at both mRNA and protein levels [54]. EMT is induced by many pathways, including TGF-β, Notch and Wnt, and Liu et al. investigated whether UCHL1 activity is involved in TGF-β and hypoxia signalling [54, 56, 57]. In the TGF-β signalling cascade, TGF-β binds to TGFβR2, triggering a phosphorylation cascade of TGFβR1 followed by SMAD2/3 (Fig. 1b). Phosphorylated SMAD2/3 binds SMAD4 and the protein complex translocates to the nucleus to regulate target gene transcription [58]. Although TGF-β acts as tumour suppressor at the early stage of cancer, it becomes an oncogenic factor at advanced stages by triggering cell invasion and stimulating cancer cell intravasation to proximal tissues, and extravasation into distal tissues [59]. Hence, TGF-β signalling plays a crucial role in breast cancer metastasis and progression and has been demonstrated to promote breast cancer metastasis in vivo [60, 61]. Using genetic approaches the authors found that UCHL1 interacts with TGFβR1 and SMAD2/3 through its N-terminal region and rescues these proteins from ubiquitination and proteasomal degradation, thereby acting as a regulator of TGF-β signalling [54]. UCHL1 has previously been suggested to regulate protein kinase B (AKT) and hypoxia-inducible factor 1 α (HIF1α) expression and signalling [62, 63]; however, Liu et al. demonstrated that expression of AKT and HIF1α were unaffected by UCHL1 knockdown in their model system [54].

**Fig. 2:**
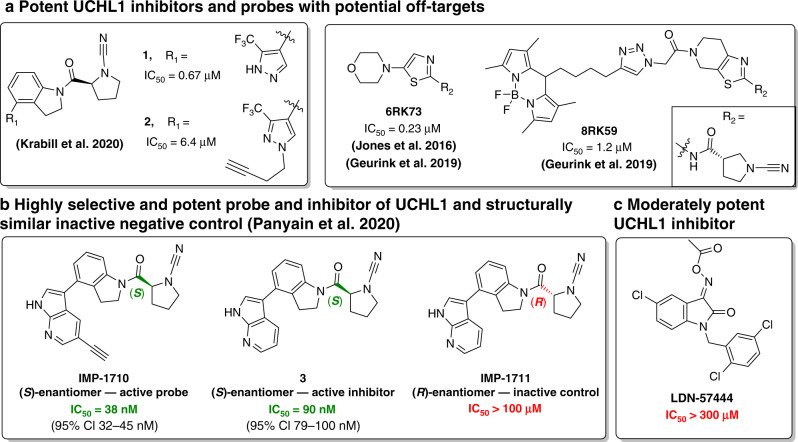
Structures and potencies of small-molecule UCHL1 inhibitors and activity-based probes (ABPs). **a** Potent UCHL1 inhibitors, ABPs and less specific compounds with potential off-targets; IC_50_ were determined by monitoring cleavage of rhodamine110 from a ubiquitin substrate (Ub-Rho) for compound **1** and **2** [82], and by cleavage of Rho-morpholine from Ub-Rho-morpholine substrate for **6RK71** and **8RK59** using fluorescence intensity assay; [64]. **b** Structures of highly selective and potent UCHL1 inhibitor **3** ((*S*)-enantiomer) and ABP **IMP-1710** ((*S*)-enantiomer) and structurally similar inactive inhibitor **IMP-1711** ((*R*)-enantiomer, negative control); **c** Promiscuous and moderately potent UCHL1 inhibitor **LDN-57444**. IC_50_ of **3**, **IMP-1710**, **IMP-1711** and **LDN-57444** were determined by a fluorescence polarisation assay using a ubiquitin substrate Ub-Lys-TAMRA [77].

To determine whether TGF-β signalling and breast cancer metastasis could be ablated by UCHL1 inhibition, Liu et al. used a covalent irreversible inhibitor **6RK73** (CAS number: 1895050-66-4), identified in a recent patent filed by Mission Therapeutics (Fig. 2a) [64, 65]. **6RK73** displayed high biochemical inhibitory potency and selectivity towards UCHL1 over other closely related DUBs, UCHL3 and UCHL5. In addition, in a TAMRA-ubiquitin-VME assay, **6RK73** showed more potent intracellular UCHL1 inhibition than **LDN-57444** (CAS number: 668467-91-2), which was very poorly active, casting doubt on its validity as a UCHL1 inhibitor (Fig. 2c). **6RK73** treatment resulted in inhibition of TGFβR1-SMAD2/3-mediated TGF-β signalling and TGF-β-induced SMAD2/3-phosphorylation, and decreased TGFβR1 and SMAD protein levels. Moreover, **6RK73** exhibited a similar inhibitory effect on breast cancer extravasation when compared with UCHL1 genetic knockdown.

UCHL1 was shown to be elevated in sera of patients with TNBC compared to healthy individuals, a phenomenon previously observed by Kuan et al. in exosomes isolated from TNBC cell conditioned medium and from sera of patients with aggressive breast cancer, where they further identified a correlation with doxorubicin resistance [66]. In several systems, including a zebrafish xenograft model, the authors provide evidence that exosomes enriched in UCHL1 upregulated TGF-β signalling in receptor cells via transfer from exosomes, facilitating migration and extravasation of breast cancer cells [54]. Together, these results suggest that UCHL1 has important roles in migration and extravasation of TNBC by promoting TGF-β signalling, and its exosomal release and activity might be useful as a blood-based biomarker for diagnosis of aggressive TNBC. In a separate study Nagata et al. recently identified UCHL1 as an active DUB in lung cancer, where UCHL1 also supports TGF-β signalling by deubiquitinating and stabilising SMAD2/3 [67]. The authors showed that UBH-1 in *Caenorhabditis elegans* and its human homolog UCHL1 promotes DAF-7/ TGF-β signalling, indicating that the mechanism of TGF-β signalling is conserved among animal species [67]. In addition, TGF-β signalling is enhanced by overexpression of UCHL1 but not the mutant UCHL1-C90A, indicating that catalytic activity of UCHL1 is mandatory for enhancing TGF-β signalling.

## Towards the validation of UCHL1 as a novel drug target

### Activity-based probes (ABPs) as an enabling technology for cancer research

**Fig. 3:**
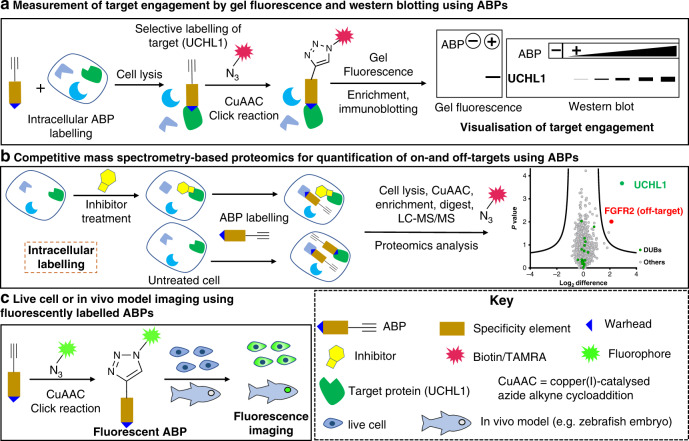
Target engagement of ABP, quantification of targets and live cell and in vivo imaging. Schematic representation of **a** measurement of target (UCHL1) engagement by gel fluorescence and western blotting using ABPs. Generally, ABP labelling is directed towards a particular target protein or protein class. ABPs may contain latent handles such as an alkyne, which may be bioorthogonally ligated with a reporter such as a fluorophore or a biotin affinity tag. If a fluorophore is used, labelled proteins may be separated by SDS-PAGE and directly visualised using fluorescence scanning, while biotinylated target proteins are generally enriched on a neutravidin-immobilised resin and visualised on western blot using target-specific antibodies; **b** quantification of on- and off-targets using ABPs and competitive mass spectrometry (MS)-based proteomics. Cells are treated with selective inhibitors/drugs, followed by intracellular labelling with ABPs, lysis, bioorthogonal ligation to biotin and enrichment of biotinylated proteins on Neutravidin-agarose resin. On-resin digestion generates peptides from probe-labelled proteins, which are subsequently analysed using liquid chromatography-mass spectrometry. Differential labelling in inhibitor-treated and untreated samples are quantified, enabling the identification of novel drug targets as well as sites of protein modification for further drug development; **c** Imaging target protein activity in live cell or in in vivo (e.g. zebrafish embryo) cancer models using fluorescently labelled ABPs and fluorescence microscopy.

The frequent disconnect between protein expression and protein activity—for example, due to post-translational activation or inhibition—presents a significant challenge to cancer biologists aiming to link genotype to phenotype, as protein activity is not easily measured by standard proteomic approaches. An activity-based probe, or ABP, is a chemical tool, which can be applied to quantify the activity of a specific enzyme, or class of enzymes, in a physiologically relevant environment independently of protein expression [68–72]. ABPs also allow monitoring of specific target engagement by inhibitors or drugs (Fig. 3a), identification of on- and off-targets (Fig. 3b) and live cell or in vivo imaging of enzyme activity or inhibition (Fig. 3c) [73, 74].

To explore DUB biology and inhibitors, broad-spectrum Ub-derived ABPs (Ub-ABPs) have been developed that covalently bind to the catalytic cysteine in the DUB active site [75]. However, these Ub-ABPs are not selective between DUBs and can only be employed in cell-free conditions due to their cellular impermeability, caused by the large Ub specificity element. As a consequence, Ub-ABPs are unable to capture dynamic DUB activity profiles that result from distinct intracellular DUB complexation and localisation [76]. To allow profiling of DUB activity and inhibition in intact cells, small-molecule ABPs with varying selectivity and potency have very recently been developed; these probes open a new window on UCHL1 activity which has the potential to validate UCHL1 as a drug target and biomarker in TNBC and ER− breast cancer.

### The first potent and selective UCHL1 inhibitors

As noted above, significant evidence now supports a role for UCHL1 as a driver of several types of breast cancers; however, this protein has until recently lacked selective and potent small-molecule inhibitors or ABPs, limiting opportunities to explore the translational potential of these findings. The only previously reported inhibitor, **LDN-57444** (Fig. 2c) has modest biochemical potency and has recently been shown to have minimal UCHL1 engagement in cells, casting doubt on experiments previously reported with this compound [64, 77, 78]. Over the past few years, small-molecule covalent UCHL1 inhibitors containing a cyanopyrrolidine moiety have been reported in the patent literature [73, 78–81]. Biochemical activity was reported for >400 small molecules using a fluorescence polarisation assay, with several exquisitely potent UCHL1 inhibitors identified (IC_50_ values ≤ 0.1 µM against UCHL1). However, no biochemical or cellular selectivity data were provided for these compounds over either other DUBs or other protein classes. Recently, papers have emerged in the peer-reviewed literature reporting covalent UCHL1 inhibitors and ABPs of varying quality based on the compounds in these patents [64, 77, 82].

In 2020, Krabill et al. investigated the mechanism of action of a previously reported cyanopyrrolidine-based covalent inhibitor **1** [80] of UCHL1 (Fig. 2) [82]. The authors confirmed that cyanopyrrolidine **1** binds to catalytic Cys90 of UCHL1 in a covalent manner, which was slowly reversible due to hydrolysis over time [82]. Employing broad-spectrum DUB ABP hemagglutinin-tagged Ub-vinylmethylester (HA-Ub-VME) in a gel shift assay, the authors observed that alongside UCHL1, compound **1** also inhibits at least one other DUB in HEK293 cell lysates, although the identity of this off-target DUB was not determined [82]. Subsequent NMR and docking studies indicated that compound **1** binds to a region overlapping ubiquitin and the UCHL1 active site groove. Compound **1** displayed growth inhibition against B cell and lung cancer cell lines (KMS11 and SW1271) possessing high endogenous UCHL1 expression and sensitivity towards UCHL1 knockdown, but also moderate efficacy against a myeloma cell line (KMS12), which has low UCHL1 expression and does not depend on UCHL1 for proliferation, confirming that this compound has important off-target effects. Indeed, a probe profiling proteomics experiment in cancer cell lines KMS11 and SW1271 using a click chemistry-enabled alkyne-tagged ABP **2** (Fig. 2, based on original inhibitor **1**) revealed a wide range of off-targets. Moreover, quantitative engagement of these off-targets relative to UCHL1 were not definitively explored, leaving the selectivity profile of these compounds undefined. Further proteomics experiments to characterise off-targets are required, particularly in competitive activity-based proteome profiling (ABPP) experiments to determine specific vs non-specific binding; however, based on the evidence reported to date these compounds lack the selectivity required to be useful as tools to study UCHL1 activity or inhibition in cells.

Fluorescent small-molecule ABPs have been recently reported to label UCHL1 in vitro, in cell lines and in vivo in zebrafish embryos by the Geurink and Ovaa labs [64]. These ABPs were designed on cyanopyrrolidine inhibitor **6RK73** reported by Mission Therapeutics [65] coupled to a range of fluorescent reporters, the most well characterised of which was BodipyFL probe **8RK59** (Fig. 2a). Importantly, the biochemical activities of fluorescent analogues were similar to the parent compound, and they efficiently labelled and inhibited recombinant UCHL1, and UCHL1 in HEK293T cell lysates. Furthermore, the authors demonstrated that **8RK59** efficiently labelled UCHL1 in intact HEK293T cells, non-small cell lung cancer (NSCLC) A549 cells, and TNBC MDA-MB-436 and SKBR7 cells [64]. They also confirmed that BodipyFL **8RK59** binds specifically to the active site Cys90 of UCHL1 using a catalytically inactive C90A mutant expressed in HEK293T cells [64]. However, **8RK59** also showed at least one other prominent off-target at 60 kDa in cells by in-gel fluorescence analysis, with a similar labelling efficiency to UCHL1.

To identify the potential off-targets of this probe, authors performed chemical proteomic experiments using azide-tagged (for click chemistry) or biotinylated ABP analogues of **6RK73**. A range of proteins from diverse classes were identified as off-targets, including Parkinson disease protein 7 (PARK7), which was labelled more than UCHL1, suggesting limited cellular selectivity of **6RK73** as a UCHL1 inhibitor. Despite this caveat and ambiguous evidence for specific UCHL1 labelling in cells by microscopy, the authors explored whether **8RK59** could visualise and track UCHL1 activity in vivo in zebrafish embryos [64]. Fluorescence imaging showed that **8RK59** was mainly localised in the eye, face and brain, which was postulated without direct evidence to be the result of a higher density of nerve cells that express UCHL1 [83].

In parallel, Panyain et al. in collaboration with Mission Therapeutics reported high quality, low nanomolar activity UCHL1 small-molecule probes and inhibitors, which target UCHL1 with minimal off-target activity, providing the most potent and selective tools currently available to probe UCHL1 in cancer [77]. Cyanopyrrolidine-based UCHL1 inhibitor **3** was elaborated from the patent literature [84] into alkyne-tagged ABP **IMP-1710** (CAS number: 2383117-96-0) (Fig. 2b). Parent inhibitor **3** and ABP **IMP-1710** delivered UCHL1 IC_50_ values of 90 and 38 nM, respectively, in a fluorescence polarisation (FP) assay using Ub-Lys-TAMRA, and displayed covalent and slowly reversible UCHL1 binding. Importantly, the (*R*)-enantiomer of this compound (**IMP-1711**) was more than 1000-fold less potent than (*S*)-enantiomer **3**, providing an effective control compound and demonstrating a specific and stereoselective interaction with UCHL1. **IMP-1710** and **3** both potently engaged UCHL1 in a breast cancer cell line (Cal51) stably expressing FLAG-tagged UCHL1 within cell EC_50_ values of 110 and 820 nM, respectively. High selectivity for UCHL1 was confirmed against a panel of 20 recombinant DUBs and in HEK293 cells, with **IMP-1710** showing concentration-dependent UCHL1 labelling with minimal or no detectable labeling of other DUBs. **IMP-1710** remained stable in media for >3 days without losing activity, and labelled UCHL1 specifically at the catalytic Cys90 in intact cells. Chemical proteomic probe profiling experiments identified only two proteins as significant targets at 20 nM **IMP-1710**: UCHL1 and fibroblast growth factor receptor 2 (FGFR2), with the former being the most enriched target. However, FGFR2 does not appear to be a physiologically significant off-target as pull-down experiments showed labelling only at very high inhibitor concentration (>1 µM). Proteome-wide competitive ABPP further confirmed that inhibitor **3** potently and selectively labels UCHL1 across in a concentration-dependent manner, in contrast to inactive control **IMP-1711** or the off-target compound **LDN-57444**.

## Discussion

### Multiple roles for UCHL1 in breast cancer

ER serves as predictive biomarker in breast cancer treatment; however, loss or reduction in ER expression makes breast cancers resistant to endocrine therapy. Multiple recent reports now support the concept that high levels of UCHL1 correlate with the suppression of ER expression [41, 49]. As noted above, Chen et al. proposed that UCHL1 deubiquitinates and stabilises EGFR, which subsequently represses ER transcription through hyperactivation of MAPK [41] and UCHL1 inhibition using **LDN-57444** or knockdown induced ER expression in ER− breast cancer cells. It should be noted, however, that LDN-57444 has been shown to have minimal UCHL1 engagement in cells, casting doubt on this specific experimental outcome. In a contrasting analysis, Liu et al. proposed that UCHL1 promotes breast cancer metastasises by deubiquitinating and stabilising TGFβR1 and SMAD2 in TNBC by maintaining the TGF-β signalling pathway [54], with UCHL1 inhibition using a cyanpyrrolidine-based covalent inhibitor **6RK73** or genetic knockdown antagonising TGF-β signalling in TNBC models. Although EMT may be induced by diverse factors including differential expression of microRNAs, TGF-β, Notch or Wnt signalling, as well as factors in the tumour microenvironment such as hypoxia, only TGF-β and hypoxia signalling have been explored to date for the role of UCHL1 in breast cancer. Further experiments will be required to investigate whether UCHL1-regulated ER status in TNBC may also involves additional pathways [56, 57]. Although **6RK73** showed more potent inhibition against UCHL1 and better cellular engagement than **LDN-57444**, it showed limited cellular selectivity with a range of off-targets, particularly against PARK7, a redox-responsive chaperone protein involved in Parkinson’s disease. It is not clear from these studies whether UCHL1 modulates ER expression and TGF-β signalling simultaneously or in a context-specific manner, as the ER expression study was conducted in ER− breast cancers, whereas the regulation of TGF-β signalling study was performed only in TNBC. Therefore, a more comprehensive set of experiments are required to explore the effect of UCHL1 inhibition in both type of cell lines (ER− and TNBC) to validate whether these roles of UCHL1 depend on cancer context. UCHL1 has been considered a cancer promoter due to its overexpression in certain type of cancers; however, its role in cancer initiation, progression and invasion are still a matter of debate. In other cancer contexts, such as ovarian [30], hepatocellular [32] and gastric cancers [85], UCHL1 DNA methylation has been reported, suggesting it may play a role in cancer suppression in these contexts. These studies point towards the conclusion that while the balance of evidence favours UCHL1 as oncogene in breast cancer, its role may be strictly cancer specific.

Despite the fact that most ER+ breast cancers initially respond well to ER modulators such as tamoxifen and fulvestrant, one third of the metastatic breast cancers eventually lose ER expression and become resistant to hormone therapy [6, 41, 86]. Therefore, UCHL1 may serve as new oncogenic target for the treatment of ER−, TNBC and ER+ breast cancer that is resistant to endocrine therapy, or potentially as an adjuvant target to supress recurrence. Although the aforementioned reports suggest that EGFR, TGFβR1 and SMAD2 are UCHL1 substrates in ER− and TNBC, unbiased proteome-wide UCHL1 substrate identification in specific disease models will be required to establish the full spectrum of UCHL1 activity and phenotypes. While more robust evidence will be required to underpin UCHL1 as a clinical biomarker and therapeutic target the data presented above provides the impetus for further studies to understand the role of UCHL1 in preclinical models and available clinical datasets. Availability and development of high-quality chemical probes as described below will provide essential tools for these studies.

### The importance of high-quality chemical probes in cancer, mechanism of action studies and drug discovery

**LDN-57444** has been widely used to inhibit UCHL1 in several type of cancers in which UCHL1 may be expressed, such as in oral squamous cell carcinoma (OSCC) [87], neuroblastoma [88, 89], non-small cell lung cancer (NSCLC) [90] and invasive carcinomas [87]. Beyond cancer, it has been used in an attempt to target or inhibit UCHL1 in numerous disease models including for Parkinson’s disease [91–93], Alzheimer’s disease [94], spinal muscular atrophy (SMA), chronic liver disease [95], inflammatory disease [96], atrial fibrillation [97], cardiac disease [98–100] and lung injury [101]. **LDN-57444** has also been employed as a tool compound to study cellular processes such as mitosis [102] and synaptic remodeling [103], and as part of a biosensor for UCHL1 detection in body fluids [104]. Troublingly, in view of the limitations of this compound, many of these papers have served as the basis for follow up studies, and have been cited widely. In cancer-related studies, **LDN-57444** was used to identify a role for UCHL1 as a prognostic marker in neuroblastoma cell differentiation, and there have even been attempts to formulate this compound to improve its aqueous solubility in order to treat invasive carcinomas [87]. The recent data discussed above suggesting that **LDN-57444** is insufficiently potent or selective to be useful as a UCHL1 probe raises questions around the robustness of these studies [77]. To further complicate matters, **LDN-5744** contains an N-oxime chemical moiety, which is known to be unstable at mild acidic or physiological pH, confounding the interpretation of experiments using this compound [105].

Successful drug discovery often relies on high-quality chemical probes to validate biological targets. The implementation of poor-quality chemical probes such as **LDN5744** can very easily lead to misleading outcomes in this context. Although they continue to be incorrectly applied or ignored across a wide range of biological studies, the criteria for defining a high-quality chemical probe have been established for some time [106], particularly in cancer [107]. Detailed and authoritative information and resources for chemical probes can be found in several freely available online databases (https://chemicalprobes.org, https://opnme.com/molecules, https://probeminer.icr.ac.uk, https://www.probes-drugs.org). We outline below the essential criteria for a high-quality chemical probe based both on our own experience and extensive literature evidence, to illustrate how two of the claimed UCHL1 probes discussed above (**IMP-1710** and **LDN-57444**) stand up against these criteria (Table 1). In summary, a high-quality probe needs to possess experimentally proven high potency, selectivity, cellular efficacy and permeability in order to constitute a useful tool to test biochemical assumptions and to validate novel therapeutic targets [76, 108].

**Table 1:** Guidelines for a high-quality chemical probe, and evaluation of **IMP-1710** and **LDN-57444** against these criteria (✓ meets requirement, ✗ does not meet requirement, − unknown as to whether meets requirement).

Parameter	Value	Typical assay/analysis format	IMP-1710	LDN-57444
Potency	IC_50_/K_d_ < 100 nM	In vitro biochemical assay	✓	✗
Selectivity over next most engaged target(s)	>30 fold	In vitro biochemical assay, proteomics analysis	✓	✗
On-target cellular activity	IC_50_ or EC_50_ < 1 µM	Cell-based assay	✓	✗
Characterisation of MoA	Inhibitor, allosteric inhibitor, agonist, irreversible inhibitor, degrader, etc	Cell-based or cell-free assay in a dose-dependent manner.	Covalent, slowly reversible	Reversible
Off-target specificity	Minimal or no off-target	In vitro biochemical assay, proteomics analysis	✓	−
Chemical stability	>90% at time point >treatment time	Time course experiment	✓	−
Quantitative effect	Dose-dependent labelling	Cell-based or in vitro assay	✓	✗
Structurally related negative control available (e.g. inactive enantiomer)	In vitro potency and cell-based activity <100 times than the active probe	In vitro assay and cellular assay	✓	✗

**IMP-1710** serves as a high quality and currently commercially available chemical probe to study UCHL1 not only within a breast cancer context, but also wherever UCHL1 has been implicated in a disease or any mechanistic studies. For instance, **IMP-1710** could be useful to study the role of UCHL1 in other cancer contexts where UCHL1 is known to regulate EGFR expression, such as in EGFR+ colorectal cancers [109], drug-resistant breast cancer [43] and thyroid and glioma carcinomas [110]. Similarly, **IMP-1710** could be employed as a tool to study UCHL1-mediated TGF-β signaling in non-cancer contexts, such as cardiac remodeling [99]. By the same token, cancers that are sensitive to UCHL1 inhibition, but in which the phenotypic outcome or mechanism of action is postulated to be orthogonal to breast cancer, could also benefit from studies using **IMP-1710** as a selective and potent probe. For example, UCHL1 has been recently suggested to regulate the expression of programmed cell death-ligand 1 (PD-L1) in the AKT-P65 signaling pathway within the context of non-small-cell lung cancer (NSCLC) [111].

### Future directions for UCHL1 as a target in breast cancer

Growing evidence demonstrates that high UCHL1 activity in specific classes of breast cancers, including ER− breast cancer and TNBC, can be targeted to enhance the efficacy of endocrine therapy in ER− breast cancer cells, as well as to mitigate TNBC migration and metastasis [41, 54]. Schroder et al. have also shown that high UCHL1 expression is negatively correlated with ER and progesterone receptor (PR) expression, and positively correlated with advanced tumour stage and shorter overall survival times for breast cancer patients (Fig. S1), further supporting a prominent role for UCHL1 in breast cancer progression [49]. Consequently, UCHL1 may serve as disease biomarker as well as a potential novel therapeutic target for breast cancers with loss or reduction of ER expression; however, it may not be a suitable target for HER2+ breast cancers, which typically have low endogenous UCHL1 expression [41]. A separate Kaplan–Meier plot analysis performed using large publicly available datasets (e.g. GEO (Affymetrix microarray only), European Genome-phenome Archive (EGA) and The Cancer Genome Atlas (TCGA)) suggested that high UCHL1 mRNA expression did not correlate with shorter survival in ER− and PR− breast cancers (Fig S2). However, any relationship to simple mRNA expression should be treated with caution, since UCHL1 transcript level may not correlate with UCHL1 protein expression, which in turn may not correlate with UCHL1 enzyme activity in the tumour. In future work, selective activity-based probes such as IMP-1710 will be powerful tools to unpack these relationships. A combination of anti-estrogen therapy (such as tamoxifen or fulvestrant) and a UCHL1 inhibitor could be a potential therapeutic strategy for ER- breast cancer, hormone-resistant breast cancers and TNBC patients. UCHL1 inhibition could also be a potential treatment for several types of other cancers where UCHL1 is frequently overexpressed, including prostate cancer [112], non-small cell lung carcinoma [113], pancreatic cancer [114], leukemia [26], colorectal cancer [33] and medullary thyroid carcinoma [115]. It will also be important for future studies to understand in detail the mechanisms by which UCHL1 expression and activity are regulated; these are currently poorly understood but may have a significant impact on the efficacy of UCHL1 inhibitors in the clinic.

To understand the involvement of UCHL1 in different cancers, future studies should look to selective and potent activity-based probes to facilitate identification of the substrates of UCHL1, and the mechanistic role played by these substrates in modulating ER expression in breast cancer, as well as in other disease contexts and in normal physiology. The combination of modulating DUB activity with proteomic ubiquitination profiling has been recently applied in other cancer contexts to identify new potential treatment paradigms. For example, Kessler and co-workers used an advanced chemical proteomics approach identify a set of oncogenic substrates of USP18 that appeared to be more susceptible to irradiation, suggesting that selective inhibition of USP18 could sensitise chronic myeloid leukaemia patients to radiotherapy [116]. Practical and robust methods to profile the substrates of UCHL1 across diverse disease contexts will be of great interest to the DUB drug discovery and chemical biology community, for which selective inhibitors such as **IMP-1710** will be powerful tools. Moreover, knowledge of the full range of UCHL1 substrates in different disease and normal physiological contexts would further assist patient stratification for UCHL1 inhibition, and help to avoid cytotoxic side effects which may arise due to undesired stabilisation of UCHL1 substrates.

## Supplementary information

Supporting Figures

## Data Availability

Not applicable.
