# Supplementary material for: UCHL1 as a novel target in breast cancer: emerging insights from cell and chemical biology
Source: Br J Cancer. 2021 Sep 8;126(1):24–33. doi: 10.1038/s41416-021-01516-5 (PMC8727673; doi:10.1038/s41416-021-01516-5)
Supplement: Supplementary file 1 — Supporting Figures [file 41416_2021_1516_MOESM1_ESM.docx]

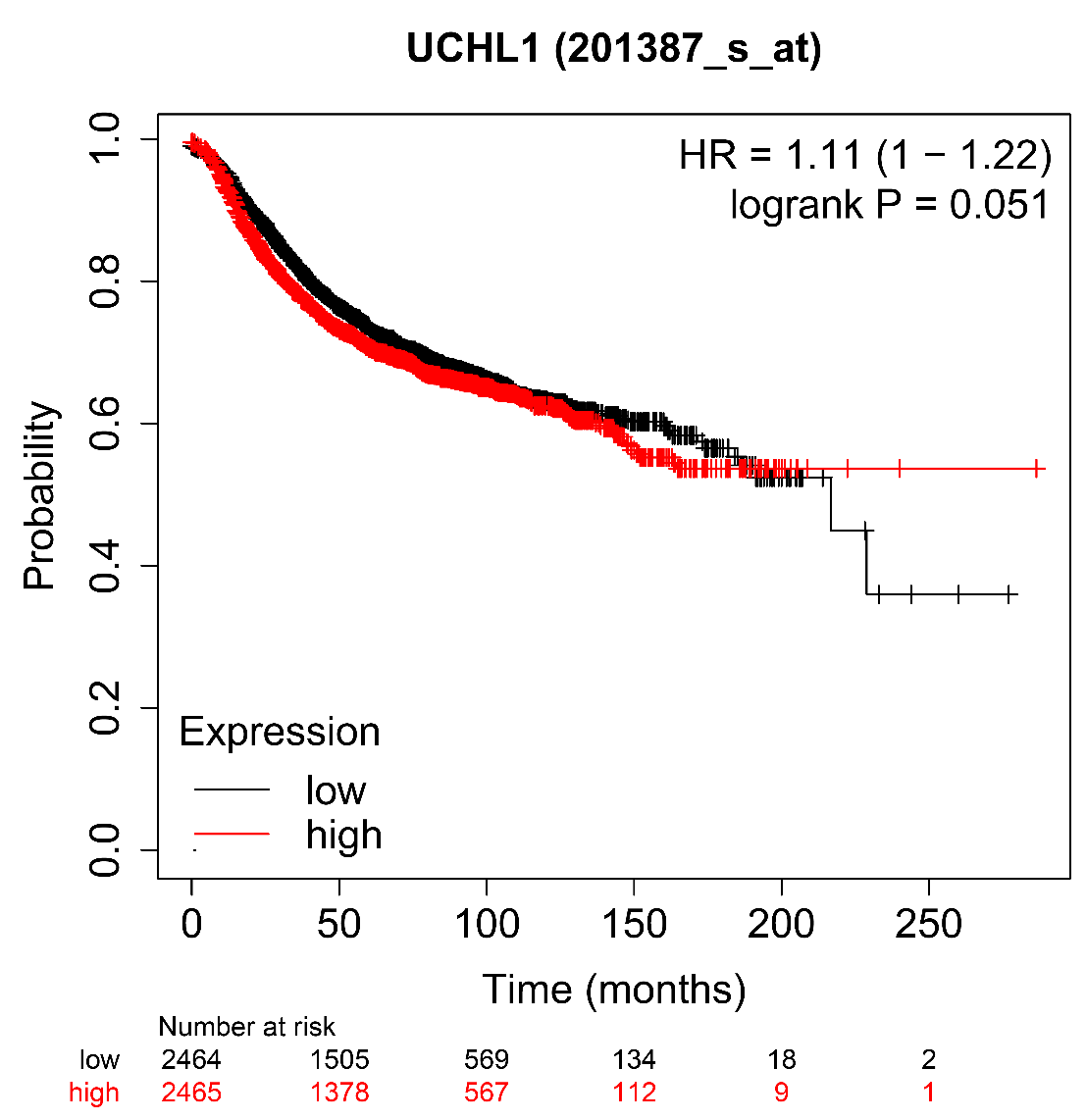


**Figure S1**: Kaplan-Meier Survival plot (KM-plotter) relationship between UCHL1 mRNA expression and overall survival in breast cancer patients (p = 0.051) using <https://kmplot.com/analysis/>. The p-value was calculated using a logrank test. The survival curve compares tumours with low (black) and high (red) expression of UCHL1.


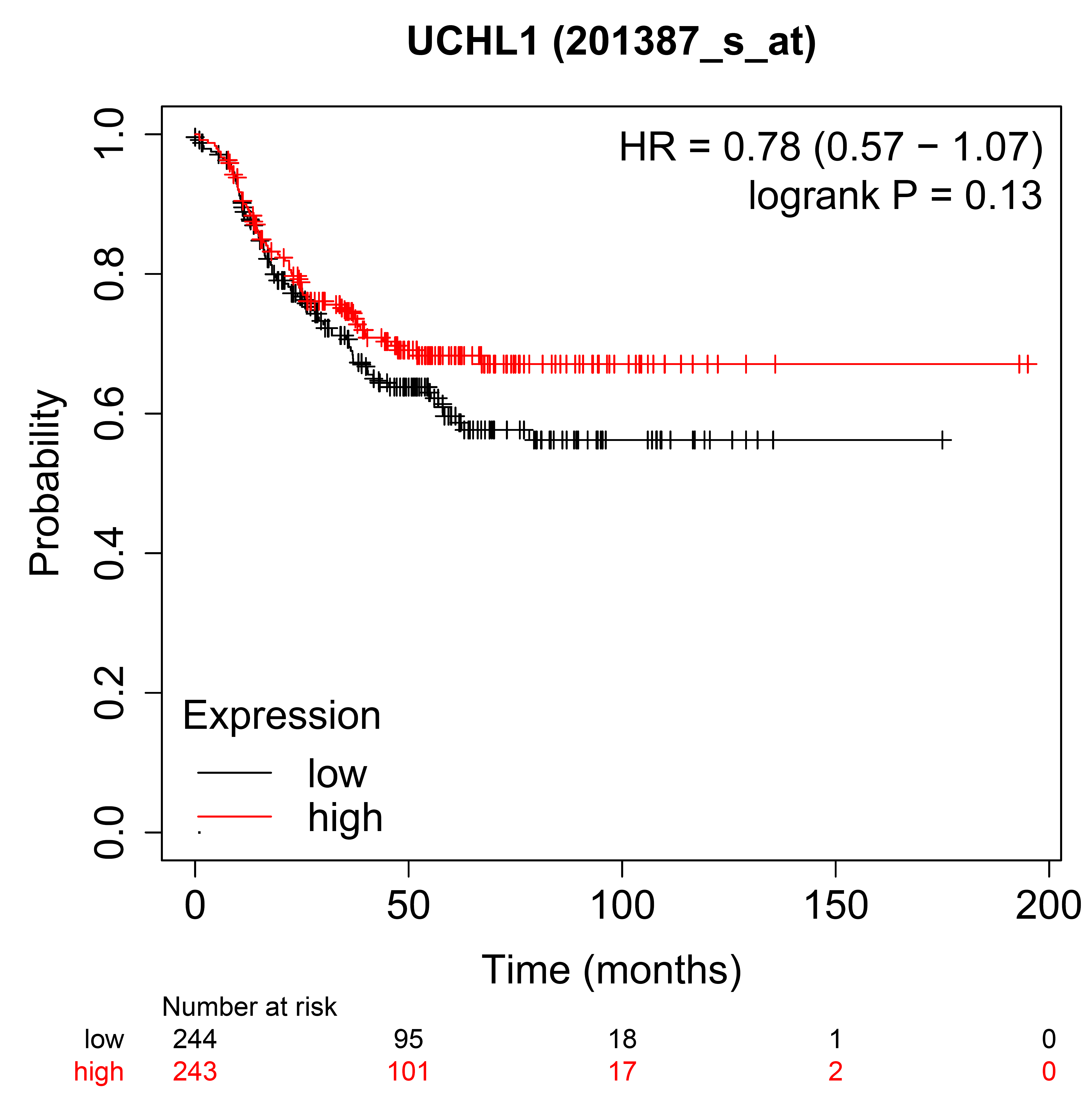


**Figure S2**: Kaplan-Meier Survival plot (KM-plotter) relationship between UCHL1 mRNA expression and overall survival in ER- and PR- breast cancer patients (p = 0.13) using <https://kmplot.com/analysis/>. The p-value was calculated using a logrank test. The survival curve compares tumours with low (black) and high (red) expression of UCHL1.
